# Serum Concentrations of IgG4 in the Spanish Adult Population: Relationship with Age, Gender, and Atopy

**DOI:** 10.1371/journal.pone.0149330

**Published:** 2016-02-24

**Authors:** Iago Carballo, Lucía Alvela, Luis-Fernando Pérez, Francisco Gude, Carmen Vidal, Manuela Alonso, Bernardo Sopeña, Arturo Gonzalez-Quintela

**Affiliations:** 1 Department of Internal Medicine, Complejo Hospitalario Universitario, Santiago de Compostela, Spain; 2 Department of Clinical Biochemistry, Complejo Hospitalario Universitario, Santiago de Compostela, Spain; 3 Department of Clinical Epidemiology, Complejo Hospitalario Universitario, Santiago de Compostela, Spain; 4 Department of Allergy, Complejo Hospitalario Universitario, Santiago de Compostela, Spain; Mario Negri Institute for Pharmacological Research and Azienda Ospedaliera Ospedali Riuniti di Bergamo, ITALY

## Abstract

**Background and Aim:**

Serum IgG4 concentrations are commonly measured in clinical practice. The aim of this study was to investigate serum IgG4 concentrations in adults and their potential relationship with demographic, lifestyle, metabolic, and allergy-related factors.

**Methods:**

Serum IgG4 concentrations were measured with a commercial assay in 413 individuals (median age 55 years, 45% males) who were randomly selected from a general adult population.

**Results:**

Median IgG4 concentration was 26.8 mg/dL. Five out of the 413 individuals (1.2%) exhibited IgG4 concentrations >135 mg/dL, and 17 out of 411 (4.1%) exhibited an IgG4/total IgG ratio >8%. Serum IgG4 concentrations were significantly higher in males than in females and decreased with age. After adjusting for age and sex, serum IgG4 concentrations were not significantly influenced by alcohol consumption, smoking or common metabolic abnormalities (obesity and the related metabolic syndrome). Serum IgG4 concentrations were not significantly correlated with serum concentrations of proinflammatory cytokines and inflammation markers. Serum IgG4 concentrations were significantly correlated with IgE concentrations. Serum IgG4 concentrations tended to be higher in atopics (individuals with IgE-mediated sensitization to aeroallergens) than in non-atopics, particularly among atopics without respiratory symptoms. Serum IgG4 concentrations were not significantly correlated with total eosinophil blood count. Cases of IgG4-related disease were neither present at baseline nor detected after a median of 11 years of follow-up.

**Conclusions:**

Studies aimed at defining reference IgG4 values should consider partitioning by age and sex. Further studies are needed to confirm the potential influence of atopy status on serum IgG4 concentrations.

## Introduction

The fourth subclass of immunoglobulin G (IgG4) is the less abundant serum IgG subclass in humans [1]. The half-life of IgG4 is approximately 21 days [2] and, unlike other IgG subclasses, IgG4 does not bind complement [3]. It is also unique among antibodies in that IgG4 molecules can be asymmetric, i.e., a large fraction of plasma IgG4 molecules have two different antigen-binding sites, resulting in bi-specificity [4,5]. Like IgE, regulation of IgG4 production is dependent on Th2 cells [5].The function of IgG4 is not completely understood. It may play a role in allergic disorders by competing for allergen with IgE bound to mast cells, thus blocking their stimulation [5]. The presence of allergen-specific IgG4, either spontaneously or after allergen immunotherapy, indicates that tolerance-inducing mechanisms have been activated [5].

Serum concentrations of IgG4 have been little studied in general populations. In a study of 172 healthy adults, French and Harrison (1984) found that IgG4 concentrations were higher in males than in females [6]. A similar finding was reported by Aucouturier et al. (1984) in a study of 173 adults [7]. No significant effect of age on IgG4 concentrations was reported in these studies, although a trend to increased concentrations in older individuals was reported [7]. To the best of our knowledge, the potential effect of common factors such as metabolic disorders (obesity and related alterations) and lifestyle factors (alcohol consumption and smoking) has been not investigated. Total serum IgG4 concentrations were not influenced by allergy, a common condition in the general population, in one study [7].

In recent decades, the methods for IgG4 measurement have improved and the interest on IgG4 concentrations has increased due to the emerging concept of IgG4-related disease (IgG4-RD), which integrates a wide number of old diseases from a new perspective [8–11]. Inflammation and fibrosis of one or more organs, with a lymphoplasmacytic infiltrate rich in IgG4-positive plasma cells is the hallmark of IgG4-RD [8–11]. The role of IgG4 in the pathogenesis of IgG4-RD is unclear. It is not known whether IgG4 antibodies are pathogenic or represent a regulatory response to another primary autoimmune or allergic process [8–15]. While raised IgG4 concentrations alone are not diagnostic of IgG4-RD because both sensitivity and specificity are not optimal [11,16–20], they nevertheless constitute one of the classic diagnostic criteria for IgG4-RD [21] and may also be useful for the monitoring of IgG4-RD [17,18].

Determining the distribution of IgG4 concentrations in the general population is important for the interpretation of reference values in clinical practice. Studies on the general population may serve to explore the potential influence of common factors and/or disorders. Accordingly, the aim of this study was to evaluate serum IgG4 concentrations in a general adult population and their potential relationship with demographic, lifestyle, metabolic, and allergy-related factors.

## Methods

### Study population and design

This study forms part of a survey of the general adult population from A-Estrada, a municipality in north-western Spain. Detailed descriptions of methodology and population characteristics have been reported elsewhere [22]. Briefly, an age-stratified random sample (n = 720) of the adult population (>18 years) of the municipality was drawn from the Health Care Registry, which covers >95% of the population. A total of 469 individuals were studied across the period January 2000-January 2001 [22]. Frozen serum samples for IgG4 determination were available for 413 of these individuals, median age 55 years (range 18–92 years). All participants were Caucasians. A total of 186 (45.0%) were males. The source study (FIS1306/99) was reviewed and approved by the Institutional Review Board of the Complejo Hospitalario Universitario from Santiago de Compostela (Spain). The present study (PGIDIT06PXIB918313) was reviewed and approved by the Clinical Research Ethics Committee from Galicia (Spain). Written informed consent was obtained from each participant in the study, which conformed to the current Helsinki Declaration.

### Classification of alcohol consumption and smoking

Alcohol consumption was evaluated in standard drinking units [23], by summing the number of glasses of wine (~10g), bottles of beer (~10g), and units of spirits (~10g) regularly consumed per week. Individuals with an habitual alcohol consumption of 1–140 g/week were defined as light drinkers, those with an alcohol consumption of 141–280 g/week were defined as moderate drinkers, and those with an alcohol consumption >280 g/week were defined as heavy drinkers. The remainder, alcohol abstainers or very occasional alcohol drinkers, were included in the same group. Consumers of at least one cigarette per day were considered smokers. Individuals who had quit smoking during the preceding year were still considered smokers, while those who had quit more than one year prior to the study were considered ex-smokers. Individuals with an habitual tobacco consumption of 1–9 cigarettes/day were defined as light smokers, and those with an habitual tobacco consumption of >9 cigarettes/day were defined as heavy smokers.

### Definition of metabolic abnormalities

Body mass index (BMI) was calculated as weight (in kg) divided by the square of height (in metres). Following standard criteria, individuals were classified as normal-weight (<25 kg/m^2^), overweight (25–30 kg/m^2^), or obese (>30 kg/m^2^).

Metabolic syndrome was defined according to Adult Treatment Panel III criteria [24], namely: (a) abdominal obesity (waist circumference >102 cm in males or >88 cm in females); (b) hypertriglyceridaemia (fasting serum triglycerides ≥150 mg/dL); (c) low HDL-cholesterol levels (fasting HDL-cholesterol <40 mg/dL in males or <50 mg/dL in females); (d) increased blood pressure (arterial blood pressure ≥130/≥85 mmHg or current anti-hypertensive medication use); and (e) hyperglycaemia (fasting serum glucose ≥110 mg/dl or current anti-diabetic therapy). Individuals who met at least three of these criteria were classified as having metabolic syndrome [24]. Routine laboratory determinations were performed with an Olympus AU-400 analyser (Olympus, Tokyo, Japan).

### Atopy traits

Atopy, the genetic tendency to become sensitized and produce IgE antibodies in response to ordinary exposures to allergens via mucosal membranes, is conventionally defined as a positive allergen-specific serum IgE or skin prick test (SPT) to any food or inhalant allergen [25]. As a consequence, these persons can develop typical symptoms of asthma, rhinoconjunctivitis, or eczema [25]. In this study, we systematically investigated allergic sensitization to inhalant allergens (aeroallergens) by means of specific IgE and SPT. Respiratory symptoms were assessed by questionnaire. In addition, the clinical records were reviewed for the presence of food allergies, allergy to drugs and Hymenoptera venoms, and additional hypersensitivity diseases such as contact dermatitis.

#### Skin prick tests (SPTs)

All subjects underwent SPTs with a panel of relevant aeroallergens in the area. The panel included mites *(Dermatophagoides pteronyssinus*, *Lepydoglyphus destructor*, and *Tyrophagus putrescentiae)*, pollens (*Lolium perenne*, *Plantago lanceolata*, *Betula alba*, and *Parietaria judaica*), moulds (*Alternaria alternata*, *Aspergillus spp*., *Penicillium notatum*, and *Cladosporium herbarum*), and animal danders (dog and cat) (ALK-Abelló, Spain). Weals >3 mm after 15 minutes were considered positive. Histamine (10 mg/mL) and normal saline were used as positive and negative controls, respectively. A detailed description of the SPT profile in this study population has been reported elsewhere [26]. The presence of at least one positive SPT was deemed to be indicative of allergic sensitization to aeroallergens.

#### Aeroallergen-specific IgE

The UniCAP-Phadiatop^™^ test (Thermo Fisher Scientific, formerly Phadia, Uppsala, Sweden) was performed on all participants. This serum test is based on the ImmunoCAP system, and consists of a solid-phase immunoassay for specific IgE using a balanced mixture of relevant aeroallergens causing common allergic respiratory disease. Calculation of results was performed automatically, by reference to the fluorescence response obtained for patient samples compared to the response obtained for the reference serum supplied. The test gives a qualitative result, which is either positive or negative depending on the fluorescence response. When a serum sample gives a fluorescence response higher than or equal to that of the reference serum, the test is interpreted as positive and indicative of allergic sensitization. A previous description of Phadiatop^™^ positivity in this population and its relationship to positive SPTs has been described elsewhere [27].

#### Respiratory symptoms

The lifetime history of respiratory symptoms was investigated at baseline in all participants by means of the following questions: (a) “have you ever had a problem with sneezing or a runny or blocked nose when you did not have a cold or the flu?”, and (b) “have you ever had wheezing or whistling in the chest at any time in the past?”, thereby exploring the presence of nasal and bronchial symptoms, respectively. Subjects who answered “yes” to either of these questions were classified as symptomatic, as reported elsewhere [26].

### Additional immune diseases

The clinical records of all participants were retrospectively searched for the presence of IgG4-RD at baseline. These records were reviewed again in November 2011 for new-onset immune disease and mortality causes. Two individuals were lost to follow-up. A total of 81 individuals died during the study period. Median follow-up was 137 months (range, 2–142 months).

### Serum immunoglobulin assays

Serum samples were stored frozen until use. The IgG4 subclasses were measured by turbidimetry using commercially available kit (The Binding Site Limited, Birmingham, UK) in a SPAPLUS^™^ analyser (The Binding Site) in strict accordance with the manufacturer's instructions. IgG4 antigen concentration was measured by turbidimetric methods using latex-enhanced antibodies to increase the relative light-scattering signal of the antigen-antibody reaction, increasing the sensitivity to 0.3 mg/dL. Precision studies were performed following CLSI Evaluation of Precision Performance of Clinical Chemistry Approved Guideline (CLSI Document EP5-A) over 21 working days, with two runs per day, obtaining a CV% <5.3 within run. According to the manufacturer, median serum IgG4 concentration in 30 healthy adults was 21.5 mg/dL and the 95^th^ percentile range was 3.9–86.4 mg/dL. A threshold IgG4 concentration of 135 mg/dL is commonly accepted as a criterion for IgG4-related disease [21].

Serum concentrations of total IgG, IgA, and IgM were determined by a commercial nephelometry assay using a BN-II device (Dade Behring, Marburg, Germany), and have been reported elsewhere [28]. The ratio of IgG4 (mg/dL) to total IgG (mg/dL) was calculated and expressed as a percentage. A threshold IgG4/IgG ratio of 8% has been proposed as a criterion for IgG4-related disease [18].

Serum concentrations of total IgE were determined by a commercial chemiluminescent enzyme immunoassay (Immulite, Siemens Medical Solutions, Gwynedd, UK), as reported elsewhere [22]. For some analyses, we calculated the ratio of total IgG4 (mg/dL) to total IgE (IU/mL).

### Additional laboratory determinations: inflammatory markers

Serum concentrations of proinflammatory cytokines (IL-6, IL-8 and TNF-alpha) were determined by a commercial chemiluminescent enzyme immunoassay (Immulite^™^), as reported elsewhere [28–30]. Serum concentrations of lipopolysaccharide binding protein (LBP) in this population were measured by the same method, as reported elsewhere [31]. Serum concentrations of the soluble form of CD14 (sCD14), an additional acute phase marker that increases after endotoxin (lipopolysaccharide) exposure, were measured by means of a commercial enzymoimmunoassay (Quantikine, R&D Systems, Minneapolis, USA) [31]. Serum concentrations of tryptase, a marker of mast cell burden and/or activation, were determined with the ImmunoCAP assay (Thermo Fisher Scientific), as reported elsewhere [32]. Blood eosinophil counts were measured in an Olympus Argos/5 Diff (ABX Diagnostics, Hamburg, Germany) automated haematology analyser.

### Statistical analyses

All analyses were performed using the Stata software package version 10 (Stata Corp, College Station, TX, USA), the SPSS software package (SPSS Inc, Chicago, IL, USA), and R-package version 3.1.2. We used the Mann-Whitney test, Kruskal-Wallis test and Jonckheere-Terpstra test (for trend analysis) for comparison of quantitative variables, and Spearman's rank test to assess correlation. Multivariate analyses were performed using linear regression, with serum IgG4 levels (log_10_-transformed to normalise their distribution) as the dependent variable. For that purpose, the only case with undetectable concentrations was attributed an arbitrary value of 0.4 mg/dL. For covariates, age (in years) was introduced into the equation as a quantitative variable, and binary variables entered the equation as ‘1’ (‘present’ or ‘yes’) or ‘0’ (‘absent’ or ‘no’). Variables were forced to enter the equation in all models. To account for stratified sampling, a design-based analysis including compensatory weights was performed for the estimation of IgG4 levels in the overall population. Centile curves for describing the distribution of IgG4 in relation to age and gender were generated using a combined method based on the Cole-Green and Rigby-Stasinopoulos [33,34] algorithm using the GAMLSS package for R. For all analyses, two-tailed *P*-values lower than 0.05 were considered statistically significant.

## Results

### Overall distribution of serum IgG4 concentrations in the population

**Fig 1:**
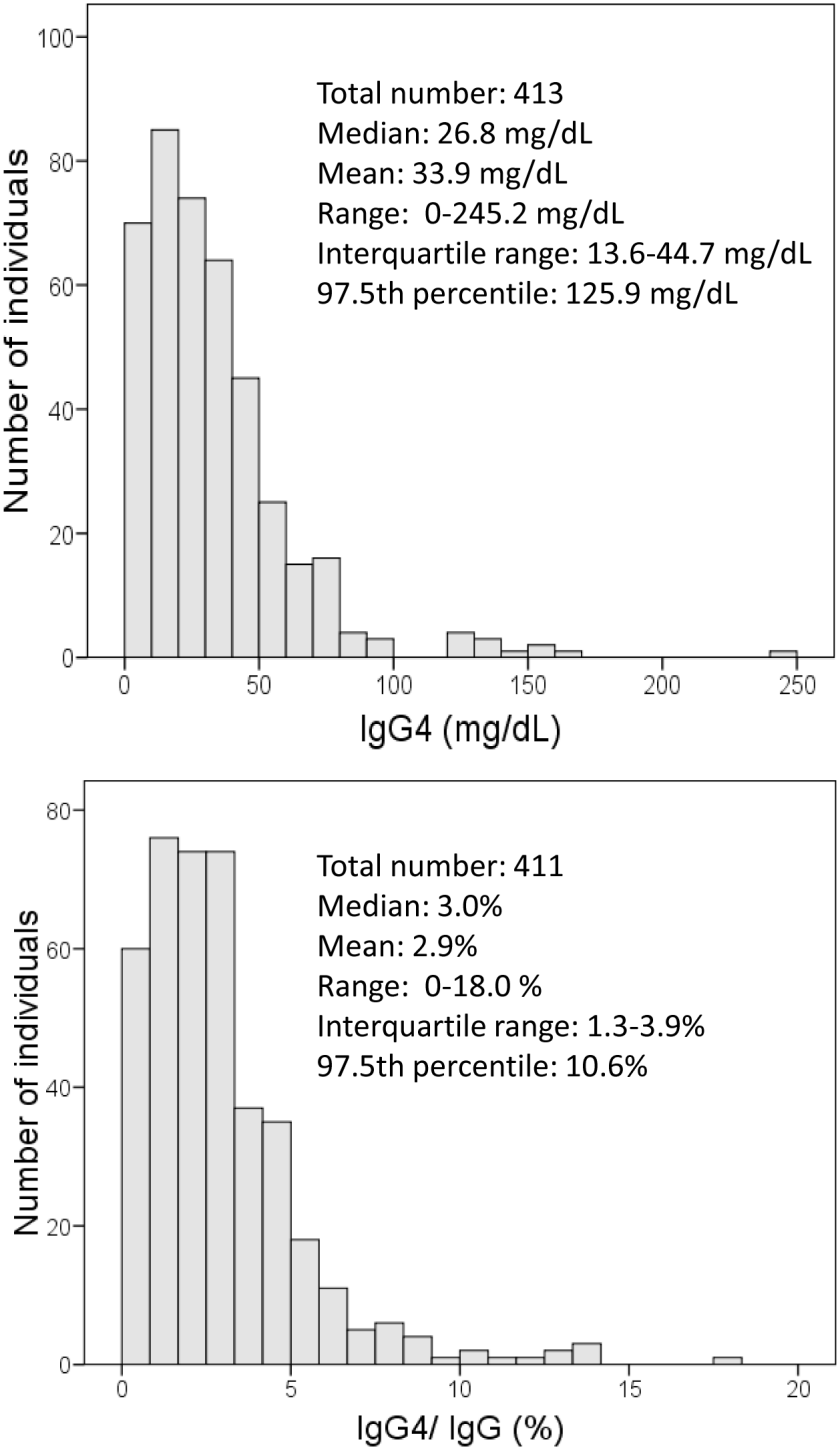
Histogram of serum igG4 concentrations and proportion of IgG4 over total IgG. Estimates (mean, median, and percentiles) were weighted according to the study design.

The histograms of serum IgG4 concentrations and IgG4/IgG ratio are represented in Fig 1. The distribution of both IgG4 and the IgG4/IgG ratio was not normal but skewed to the right (Fig 1). Five individuals (1.2%) showed IgG4 concentrations higher than 135 mg/dL, and 17 individuals (4.1%) showed an IgG4/IgG ratio higher than 8%.

### Relation of serum IgG4 concentrations to age and sex

**Fig 2:**
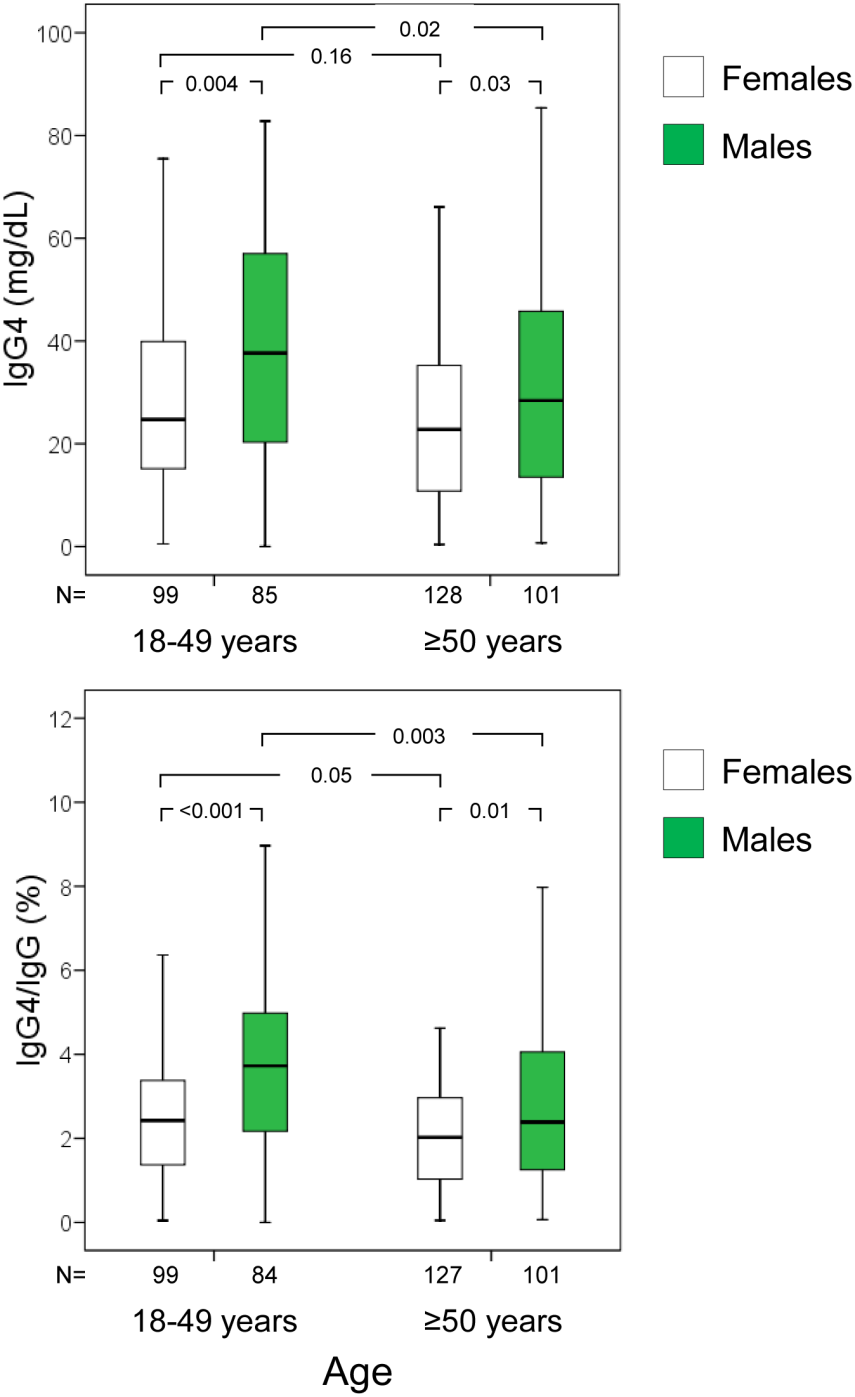
Boxplots of serum igG4 concentrations and the proportion of IgG4 over total IgG in study participants, stratified by age and sex. Outliers (values laying more than one and a half times the length of the box [interquartile range] from either end of the box) are not shown but are included in analyses.

**Fig 3:**
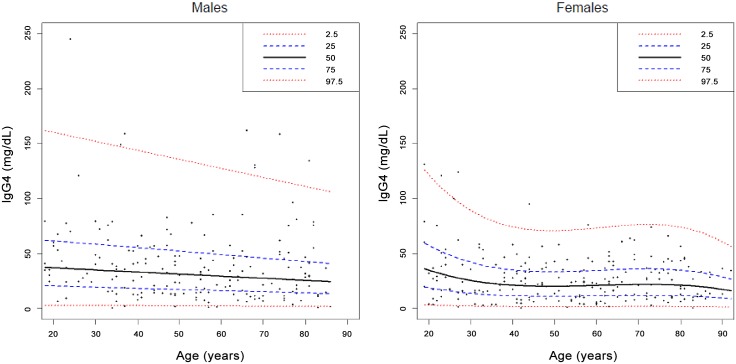
Percentiles (2.5, 25, 50, 75, and 97.5th) of serum IgG4 concentrations in relation to age in males and females. Centiles were calculated using the Cole-Green and Rigby-Stasinopoulos [33,34] algorithm.

Serum IgG4 concentrations and the IgG4/IgG ratio were higher in males than in females (Table 1), and tended to decrease with age (P for trend 0.009 and <0.001, respectively). After stratification for decades of age, the decline in serum IgG4 and the IgG4/IgG ratio was particularly evident from age 50 years onwards (Table 1) in males and females alike, though it was more evident in males (Fig 2). Median IgG4 difference between individuals younger and older than 50 years was 6.1 mg/dL (95% confidence interval, 0.1–11.9 mg/dL). Median IgG4 difference between males and females was 8.6 mg/dL (95% confidence interval, 3.3–13.6 mg/dL). In the multivariate analyses (linear regression), younger age and male gender were independently associated with serum IgG4 concentrations (Table 2). A detailed analysis of IgG4 centiles in relation to age in males and females is presented in Fig 3.

**Table 1:** Serum IgG4 concentrations and proportion of IgG4 over total IgG in relation to demographic, lifestyle, metabolic, and atopic factors.

Factor	No.	IgG4 (mg/dL)	No.	IgG4/IgG (%)
**Gender**				
Female (reference)	227	23.4 (11.9–38.2)	226	2.1 (1.1–3.2)
Male	186	32.0 (14.4–51.6)***	185	2.9 (1.6–4.6)***
**Age** (years)				
18–30 (ref.)	61	31.5 (16.8–58.5)	61	2.9 (1.6–5.2)
>30–40	53	29.0 (15.7–50.4)	53	3.0 (1.6–4.4)
>40–50	70	28.3 (13.6–43.3)	69	2.6 (1.3–3.7)
>50–60	61	23.7 (11.4–34.9)*	60	2.1 (1.3–3.4)**
>60–70	67	24.5 (11.9–42.5)	67	2.2 (1.1–3.5)*
>70–80	56	26.2 (11.1–42.9)	56	2.2 (1.0–3.3)*
>80	45	20.1 (11.4–37.5)*	45	1.9 (1.1–3.0)**
**Alcohol consumption**				
Abstainers (ref.)	193	27.7 (14.3–45.2)	193	2.5 (1.4–3.9)
Light drinkers	131	23.8 (12.4–41.0)	129	2.2 (1.2–3.6)
Moderate drinkers	52	26.3 (12.1–42.1)	52	2.5 (1.3–3.9)
Heavy drinkers	37	32.4 (16.0–48.3)	37	2.9 (1.4–4.3)
**Smoking**				
Never smokers (ref.)	260	24.4 (12.2–40.3)	259	2.1 (1.2–3.3)
Ex-smokers	63	26.2 (14.0–46.1)	63	2.5 (1.3–3.9)
Light smokers	20	29.6 (13.4–44.3)	20	2.8 (1.1–4.0)
Heavy smokers	70	33.2 (20.1–51.7)**	69	3.2 (2.0–4.7)***
**Body mass index**				
Normal weight (ref.)	112	29.0 (15.7–44.0)	112	2.8 (1.6–4.1)
Overweight	179	25.6 (12.8–45.1)	177	2.4 (1.3–3.9)
Obese	121	25.3 (13.1–42.5)	121	2.2 (1.3–3.7)
**Metabolic syndrome**				
No (ref.)	313	26.9 (13.3–45.4)	311	2.5 (1.3–4.0)
Yes	100	25.0 (13.1–39.8)	100	2.2 (1.3–3.5)
**Specific IgE**				
Negative (ref.)	308	24.9 (12.7–42.0)	306	2.3 (1.2–3.7)
Positive	105	33.2 (17.3–47.2)*	105	2.8 (1.5–4.3)*
**Skin prick tests**				
Negative (ref.)	302	26.2 (12.9–41.7)	300	2.4 (1.2–3.8)
Positive	111	28.5 (16.2–46.7)	111	2.6 (1.5–4.4)

Data are expressed as medians and interquartile (25^th^-75^th^ percentile) ranges (within parentheses).

*P<0.05,

**P<0.01,

***P<0.001 with respect to the reference category (ref).

**Table 2:** Multivariate analyses (linear regression) of factors associated with serum IgG4 concentrations.

	Unadjusted		Age- and sex-adjusted	
Factor	B-coefficient (SE)	P-value	B-coefficient (SE)	P-value
**Gender** (0:female; 1: male)	0.130 (0.041)	0.002	0.129 (0.041)	0.002
**Age** (years)	-0.002 (0.001)	0.024	-0.002 (0.001)	0.023
**Heavy drinking** (0:no; 1: yes)	0.040 (0.073)	0.583	-0.051 (0.077)	0.504
**Current smoking** (0:no; 1: yes)	0.094 (0.050)	0.061	0.021 (0.056)	0.709
**Obesity** (0:no; 1: yes)	-0.030 (0.046)	0.516	-0.014 (0.045)	0.754
**Metabolic syndrome** (0:no; 1: yes)	-0.038 (0.049)	0.435	-0.010 (0.049)	0.845
**sIgE** (0: negative; 1: positive)	0.095 (0.048)	0.046	0.067 (0.048)	0.161
**SPT** (0: negative; 1: positive)	0.069 (0.047)	0.141	0.045 (0.048)	0.347

Serum IgG4 concentrations were log_10_-transformed in order to normalize their distribution. Data are expressed as slope (B-coefficient) and standard error (within parentheses). SPT, skin prick tests, sIgE, specific IgE (Phadiatiop^™^ test).

### Relation of serum IgG4 concentrations to lifestyle variables

Serum IgG4 concentrations and the IgG4/IgG ratio were similar in alcohol abstainers and alcohol drinkers (Table 1). In the univariate analyses, serum IgG4 concentrations and the IgG4/IgG ratio were higher in heavy smokers than in the reference category (never smokers, Table 1). However, the association of smoking with serum IgG4 was greatly attenuated after adjusting for age and sex in the multivariate analyses (Table 2).

### Relation of serum IgG4 concentrations to metabolic abnormalities

Serum IgG4 concentrations and IgG4/IgG ratio were not significantly associated with overweight, obesity, the metabolic syndrome (Table 1) or its components (data not shown).

### Relation of serum IgG4 concentrations to atopy traits

**Fig 4:**
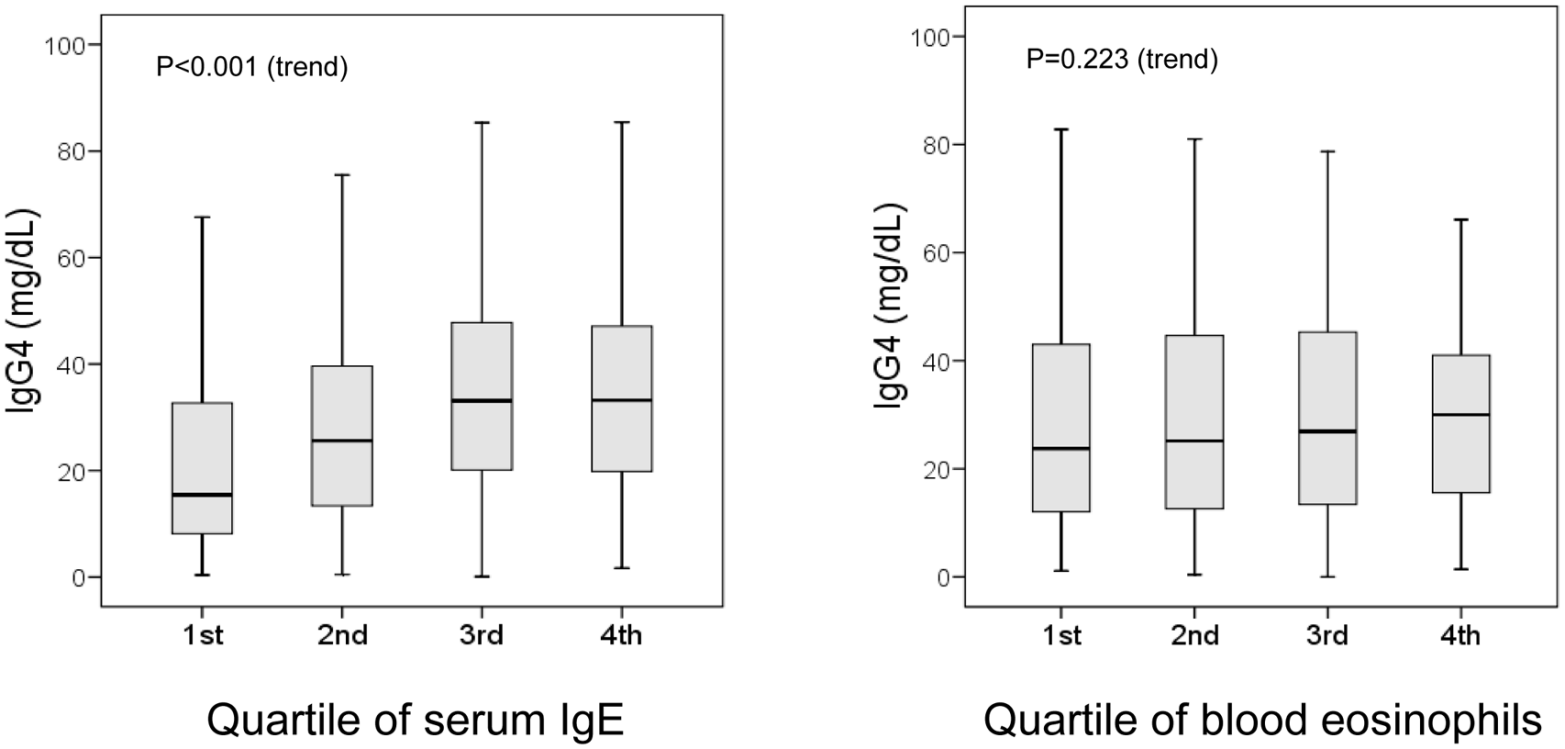
Boxplots of serum IgG4 concentrations in relation to quartiles of total serum IgE and total blood eosinophil count. Outliers (values laying more than one and a half times the length of the box [interquartile range] from either end of the box) are not shown but are included in analyses. IgG4, IgE, and eosinophil count data are available for all 413 participants.

There was a significantly positive correlation between serum IgG4 and serum IgE concentrations (Fig 4). On the contrary, there was no significant correlation between serum IgG4 concentrations and total eosinophil count (Fig 4).

In the univariate analyses, serum IgG4 concentrations and the IgG4/IgG ratio were higher among individuals who tested positive aeroallergen-specific serum IgE (Phadiatop test) (Table 1). Similar results when individuals were further stratified by SPT results (Table 3). There was no significant association between serum IgG4 concentrations and SPT positivity to aeroallergens (Table 1). The association was more evident when SPT-positive individuals were stratified by presence or absence of respiratory symptoms (Table 3). Asymptomatic, SPT-positive individuals showed the highest IgG4 concentrations, which were higher than those of SPT-negative (Table 3) and symptomatic SPT-positive individuals (P = 0.01). The association between these atopy traits and serum IgG4 concentrations was attenuated after adjusting for age and sex (Table 2). Significant differences were observed among these groups defined by SPT-positivity and respiratory symptoms in terms of the IgG4/IgE ratio (Table 3). The highest IgG4/IgE ratio was observed among SPT-negative individuals and the lowest IgG4/IgE ratio was observed among symptomatic SPT-positive individuals (Table 3). Asymptomatic SPT-positive individuals showed a significantly higher IgG4/IgE ratio than did symptomatic SPT-positive individuals (P = 0.001, Table 3). Differences in the IgG4/IgE ratio among groups, however, were largely dependent on variation of serum IgE concentrations in these groups (Table 3).

**Table 3:** Concentrations of serum IgG4, total serum IgE, and their ratio in relation to atopy traits.

Factor	No.	IgG4 (mg/dL)	IgE (kU/L)	IgG4/IgE ratio
**SPT/sIgE positivity**				
SPT-negative, sIgE negative (ref)	273	26.0 (12.8–42.1)	34.9 (14.8–71.9)	0.69 (0.30–1.36)
SPT-negative, sIgE positive	29	33.2 (14.1–42.7)	114 (66.5–452)**	0.19 (0.11–0.34)**
SPT-positive, sIgE negative	35	22.0 (10.9–43.1)	89.6 (42.6–156)**	0.31 (0.08–0.57)**
SPT-positive, sIgE positive	76	33.2 (18.3–47.7)*	213 (125–463)**	0.10 (0.04–0.37)**
**SPT/respiratory symptoms**				
SPT-negative, asymptomatic (ref)	172	27.6 (13.2–45.9)	37.9 (17.1–86.0)	0.61 (025–1.36)
SPT-negative, symptomatic	130	24.0 (12.8–36.8)	42.3 (15.5–84.5)	0.56 (0.28–1.15)
SPT-positive, asymptomatic	41	38.4 (21.7–56.6)*	134 (53.6–252)**	0.31 (0.10–0.67)**
SPT-positive, symptomatic	70	24.2 (12.6–42.8)	194 (109–470)**	0.10 (0.04–0.35)**

Data are expressed as medians and interquartile (25^th^-75^th^ percentile) ranges (within parentheses).

*P<0.05,

**P<0.001 with respect to the reference category.

Ref, reference category; SPT, skin prick test; sIgE specific IgE (Phadiatop^™^ test).

In addition to respiratory allergy, hypersensitivity diseases in the series included two individuals with contact dermatitis, two individuals with Hymenoptera venom allergy, and one individual with beta-lactam allergy. Serum IgG4 concentrations in these 5 cases were similar to those from the overall sample (median 24.1 mg/dL, and range 10.0–32.4 mg/dL).

### Additional immune diseases

No cases of IgG4-RD were present in the clinical records at baseline. Likewise, no cases indicative of IgG4-RD were detected during follow-up. New-onset diseases of potential immune basis included thyroid disease (n = 9), psoriasis (n = 4), rheumatoid arthritis (n = 3), ulcerative colitis (n = 2), Still´s disease (n = 1), Raynaud´s phenomenon (n = 1), autoimmune thrombopenia (n = 1), sarcoidosis (n = 1), small-vessel vasculitis (n = 1), and chronic autoimmune hepatitis (n = 1). There were no differences in baseline IgG4 concentrations between individuals who developed immune disease and those who did not (data not shown); or between individuals who died during follow-up and those who did not (data not shown).

### Relation of serum IgG4 concentrations to other immunoglobulins and inflammatory markers

Serum IgG4 concentrations were significantly correlated with those of IgG and IgA, though correlation was weak, particularly for IgA (Table 2). There was no significant correlation between serum IgG4 concentrations and serum levels of proinflammatory cytokines (IL-6 and TNF-alpha), acute phase reactants (LBP and sCD14), and mast cell tryptase (Table 4). There was a weakly significant, negative correlation between IL-8 and IgG4 concentrations (Table 4). Serum IgG4 concentrations were not significantly correlated with markers of liver or kidney dysfunction (data not shown).

**Table 4:** Correlation of serum concentrations of IgG4 and concentrations of immunoglobulins (total IgG, IgA, IgM) and inflammatory markers (mast cell tryptase, soluble CD14 [sCD14], lipopolysaccharide-binding protein [LBP], and proinflammatory cytokines [IL-6, IL-8, and TNF-alpha]).

	IgG (mg/dL)	IgA (mg/dL)	IgM (mg/dL)	Tryptase (μg/L)	IL-6 (pg/mL)	IL-8 (pg/mL)	TNF-α (pg/mL)	sCD14 (μg/mL)	LBP (μg/mL)
**Coefficient of correlation with IgG4** (mg/dL)	0.250	0.108	-0.085	-0.060	0.016	-0.118	-0.070	-0.035	-0.081
**95% confidence interval for the coefficient**	0.154; 0.343	0.003; 0.214	-0.176; 0.018	-0149; 0.039	-0.085; 0.114	-0.215; -0.016	-0.174; 0.027	-0.129; 0.068	-0.172; 0.011
**No.**	411	411	411	413	411	411	411	413	413
**P-value**	<0.001	0.03	0.09	0.22	0.75	0.01	0.16	0.48	0.10

## Discussion

This study shows that serum concentrations of IgG4 in the adult population vary with age and sex, and are higher in males and younger individuals. These differences are independent of both lifestyle (alcohol and smoking) and common metabolic abnormalities (obesity and related disorders). According to our results, median IgG4 values may be 36% higher in males than in females. This is in contrast with total IgG concentrations, which tended to be slightly higher in females than in males in the same population [28]. Sex differences in immunoglobulin concentrations, specifically higher IgM levels in females, have been attributed to hormonal effects on B lymphocytes [35,36]. Serum IgG4 concentrations decreased with age, e.g., median IgG4 values were 33% lower in elderly individuals than in those aged 18–31 years. This is also in contrast with total IgG concentrations, which increased with ageing in the same population [28]. Previous studies also reported higher IgG4 concentrations in males [6,7]. However, decreasing IgG4 concentrations with age have been not reported previously. Moreover, a non-significant trend to increasing IgG4 concentrations with ageing was reported in some studies [7], which included fewer individuals from a narrower age range than the present study. In our experience, the decrease in IgG4 concentrations with age was significant and independent of covariates. The ratio of serum IgG4 to total IgG (IgG4/IgG) showed a similar relationship with age and sex to that shown by IgG4 alone. The relationship of the IgG4/IgG ratio with these demographic variables was even more evident than that of IgG4 alone, probably because IgG4 and total IgG tend to display opposite associations with age and sex. Such sex-and-age differences may be particularly important when interpreting IgG4 concentrations in diseases that predominate in specific demographic subsets, such as IgG4-RD, which predominates in males and in individuals older than 60 years [11,12,20]. Accordingly, future studies conducted to establish IgG4 reference values should take age and sex changes into account.

Serum IgG4 concentrations were not significantly influenced by common lifestyle factors, such as alcohol consumption and smoking. This is in contrast with serum IgA and total IgG concentrations, which are respectively increased and decreased in alcohol consumers [28]. Likewise, there was no significant association between common metabolic abnormalities (obesity and the related metabolic syndrome) and IgG4 concentrations. Both obesity and metabolic syndrome are low-grade inflammatory disorders [37] that were found to be associated with increased IL-6 and IgA values in previous studies in the same population [28]. In our experience, serum IgG4 concentrations were not correlated with most markers of inflammation (with the exception of a weakly negative correlation with serum IL-8). Likewise, serum IgG4 concentrations seemed unaffected by mild liver or kidney dysfunction (data not shown).

There were some intriguing associations between serum IgG4 concentrations and allergy traits. Serum IgG4 and IgE concentrations were significantly correlated. This is consistent with a common Th2-driven stimulus for both IgE and IgG4 [4]. Furthermore, it is consistent with the frequent finding of increased serum IgE and additional Th2-driven phenomena in patients with IgG4-RD [10,11]. Serum IgG4 concentrations tended to be higher among individuals with atopy (as defined by sensitization to aeroallergens), particularly among those who were asymptomatic (i.e., with neither nasal nor bronchial symptoms) as opposed to those who were symptomatic. Moreover, symptomatic atopic individuals registered the lowest ratios of total serum IgG4 to total serum IgE (IgG4/IgE), although this was largely a consequence of increased total IgE in atopic individuals. Taken together, these results are consistent with a tolerogenic effect of IgG4 for allergic respiratory disease [4]. These results should be interpreted with caution, however, because statistical significance was attenuated after adjustment for age and sex. Furthermore, the increased IgG4/IgE ratio that is used as a clinical marker to detect the efficacy of tolerance-inducing therapies in patients with allergy [38–39] refers to specific rather than total IgG4 and IgE. Finally, the study included a systematic evaluation of IgE-mediated sensitization to aeroallergens, but the potential sensitization to different allergens was only assessed by review of clinical records. Serum IgG4 was significantly correlated neither with blood eosinophil counts nor with serum tryptase, a marker of mast cell burden or mast cell activation [32].

The strengths of the study include its: population-based design, random sampling and wide age range of participants; use of a reliable, standard method for IgG4 determination; long-term follow-up; and attempt to reduce confounding by controlling for a number of common factors. To the best of our knowledge, there have been no previous studies of a similar nature conducted on a general population. With regard to external validity, it should be noted that all participants were Caucasians and that many cases of IgG4-RD (autoimmune pancreatitis, in particular) have been reported in Asian populations [14,15,18,20]. To the best of our knowledge, no ethnic-related differences in IgG4 concentrations have been described, though this cannot be excluded.

The attention paid to serum IgG4 concentrations has increased in recent years due to the emerging concept of IgG4-RD. The present study was not conducted to investigate the diagnostic accuracy of serum IgG4 for the diagnosis of IgG4-RD. Furthermore, the study was not powered to detect the incidence of such a rare disorder [40]. The diagnosis of IgG4-RD usually requires a tissue biopsy [10,11,19,41–43]. Elevated concentrations of IgG4 are consistent with, but not diagnostic of, IgG4-RD [10,11,19,41–43]. It is recommended that patients suspected of having an IgG4-RD have their serum IgG4 concentration measured. The results of the present study may serve to better interpret serum IgG4 concentrations in clinical practice. In summary, serum concentrations of IgG4 may be influenced by age and sex, and are not significantly influenced by common factors such as smoking, alcohol consumption, obesity and metabolic syndrome. Further studies are needed to confirm the potential influence of atopy status on serum IgG4 concentrations. Future studies aimed at defining reference IgG4 values should consider partitioning by age and sex.

## Supporting Information

S1 Table Individual data. (XLS)
